# Repurposed Drugs for Heterotopic Ossification Management: Revitalizing Therapeutic Strategies

**DOI:** 10.3390/ph18111609

**Published:** 2025-10-24

**Authors:** Ana Alonso-Pérez, Eloi Franco-Trepat, María Guillán-Fresco, Miriam López-Fagúndez, Andrés Pazos-Pérez, Verónica López, Antonio Salas, Federico Martinón-Torres, Alberto A. Jorge-Mora, Rodolfo Gómez

**Affiliations:** 1 Musculoskeletal Pathology Group, Laboratory 18, Health Research Institute of Santiago de Compostela (IDIS), Santiago University Clinical Hospital SERGAS, 15706 Santiago de Compostela, Spain; ana.alonso.perez@sergas.es (A.A.-P.);; 2 Grupo de Investigación en Genética, Vacunas, Infecciones y Pediatría (GENVIP), Health Research Institute of Santiago de Compostela (IDIS), Santiago University Clinical Hospital SERGAS, 15706 Santiago de Compostela, Spain; 3 Unidade de Xenética, Departamento de Anatomía Patolóxica e Ciencias Forense, Instituto de Ciencias Forenses, Medicine School, University of Santiago de Compostela, 15782 Santiago de Compostela, Spain; 4 GenPoB Research Group, Health Research Institute of Santiago de Compostela (IDIS), Santiago University Clinical Hospital SERGAS, 15706 Santiago de Compostela, Spain; 5 Translational Pediatrics and Infectious Diseases, Department of Pediatrics, Santiago University Clinical Hospital SERGAS, 15706 Santiago de Compostela, Spain; 6 Traumatology and Orthopedics Service, SERGAS, A Coruña University Clinical Hospital, 15006 A Coruña, Spain

**Keywords:** heterotopic ossification, osteoblast, adipocyte, endochondral ossification, therapy, bone

## Abstract

**Background and Objectives**: Heterotopic ossification (HO) involves abnormal bone growth in soft tissues. Current treatments are ineffective and prone to adverse effects, suggesting the need for new HO therapies. Intramembranous bone growth is led by osteoblasts. Since osteoblastogenesis and adipogenesis are opposed and mutually controlled processes, this study aims to identify a new repurposed therapeutic tool to inhibit osteoblastogenesis through adipogenesis promotion. **Methods**: We performed docking experiments between peroxisome proliferator-activated receptor-γ and bone metabolism-affecting drugs, namely, thiazolidinediones (rosiglitazone, pioglitazone), indomethacin, and dexamethasone, to test tritherapy antiosteoblastogenic effect. Mouse mesenchymal stem cells (C3H10T1/2), human osteoblast-like cells (SaOS2 and primary preosteoblasts), and mouse chondrocytes (ATDC5) were differentiated in the presence of these compounds. The effects on osteoblastogenesis, adipogenesis, and endochondral ossification were analysed through marker gene expression via RT–qPCR. Additionally, primary human HO cells and a congenital HO patient were treated with the selected drug combination (P-tritherapy). **Results**: Tritherapy significantly and synergistically promoted the expression of an adipogenic marker (fatty acid-binding protein 4) and decreased the expression of an osteoblastogenic marker (osteopontin). In an endochondral ossification model, it reduced ossification markers (collagen-2α1) expression, and in HO cells, it increased adipogenesis markers’ expression. Clinically, P-tritherapy administration prompted bone resorption in a patient with progressive osseous heteroplasia. **Conclusions**: Tritherapy induced adipogenesis while inhibiting osteoblastogenesis and endochondral ossification, demonstrating its potential as a new therapeutic tool to prevent abnormal bone growth. These results were consistent with bone turnover modification observed in a congenital HO patient. This concordance underscores tritherapy potential for rapid and safe translation to prevent HO.

## 1. Introduction

Heterotopic ossification (HO) is the process by which bone tissue grows outside the skeleton, mainly in soft tissues. Researchers have not fully elucidated whether this bone growth occurs through intramembranous ossification or endochondral ossification. Intramembranous bone growth is generated by osteoblasts differentiated from mesenchymal stem cells (MSCs). MSCs can differentiate not only into osteoblasts but also into adipocytes, chondrocytes, myocytes, fibroblasts, or other types of cells. Interestingly, osteoblastogenesis and adipogenesis are opposite and mutually controlled processes. Thus, bone growth depends on the proper balance between these two types of MSC differentiation. An alteration of this equilibrium toward adipogenesis causes bone loss, as in osteoporosis, obesity, or ageing [1,2]. Conversely, when the balance allows for osteoblastogenesis, excessive bone growth occurs. Ossification can also happen from a cartilaginous scaffold, being called endochondral ossification. Here, the chondrocytes proliferate, condense and then differentiate into hypertrophic chondrocytes that generate a specific matrix that will be calcified. In the end, these chondrocytes are substituted by osteoblasts that create ossification centres [3]. One of the major HO proteins is osteopontin (SPP1) [4]. It is related both to osteoblastogenic–adipogenic balance (through adipogenic inhibition) [5] and to endochondral processes (inducing transforming growth factor β (TGFβ)-mediated fibrosis) [6].

In terms of its origin, HO can be either congenital or acquired. One of the clinical manifestations of congenital HO is fibrodysplasia ossificans progressive (FOP), where injured tissues are replaced with heterotopic bone, causing chronic pain and, eventually, skeletal fusion and immobilisation [7]. Another congenital HO is progressive osseous heteroplasia (POH), characterised by cutaneous or subcutaneous ossifications [8]. Acquired HO appears following a trigger such as trauma or injury. Patients with neurological injuries, large burns, electrocutions, and other traumatic events also experience abnormal bone growth [9,10]. An important percentage of acquired HO is due to joint surgical replacement. Following hip total arthroplasty or severe long bone fracture, one in every three patients will develop HO [11], even though most of them are usually asymptomatic.

Currently, there are no pharmacological approaches to treat this pathology. Nonsteroid anti-inflammatory drugs (NSAIDs), such as indomethacin [12] and ibuprofen [13], are considered HO treatments. These molecules have been shown to prevent HO recurrence after total hip replacement [12,13] but not in all patients. Although bisphosphonates have also been considered, there is not enough evidence to support their use [13]. With respect to nonpharmacological treatments, surgical removal is an option (depending on ectopic bone localization), although it has been associated with a high relapse rate [7]. Currently, radiotherapy is one of the most commonly employed treatments [14]. It has been used as a prophylactic measure since the Seventies for concrete situations such as knee [15], hip [16], elbow [17], or vertebral [16] HO. Even though the benefit–risk ratio for a radiotherapy dosage has been approved, it is not a suitable treatment for the paediatric population [18]. Indeed, despite its effects on the HO recurrence ratio, it is considered an aggressive and unspecific treatment. Conservative treatments, such as physiotherapist mobilisation techniques, have also been considered therapeutic tools to prevent HO [19].

Currently available therapeutic tools to prevent HO have shown only partial results, and those with the strongest effects are highly aggressive for the target patients. Therefore, finding an effective and nonaggressive treatment to prevent ectopic bone formation is a clinical need. It should be considered that systemic administration of bone metabolism modifying drugs can affect overall skeletal health. These effects underscore the need to consider any potential systemic effect when developing a new therapy for HO.

In this work, we propose a new therapy to prevent and treat HO through the control of both intramembranous and endochondral ossification and the regulation of the osteoblastogenic–adipogenic balance.

## 2. Results

### 2.1. Selection of Repurposed Drugs to Block Bone Anabolism

**Figure 1:**
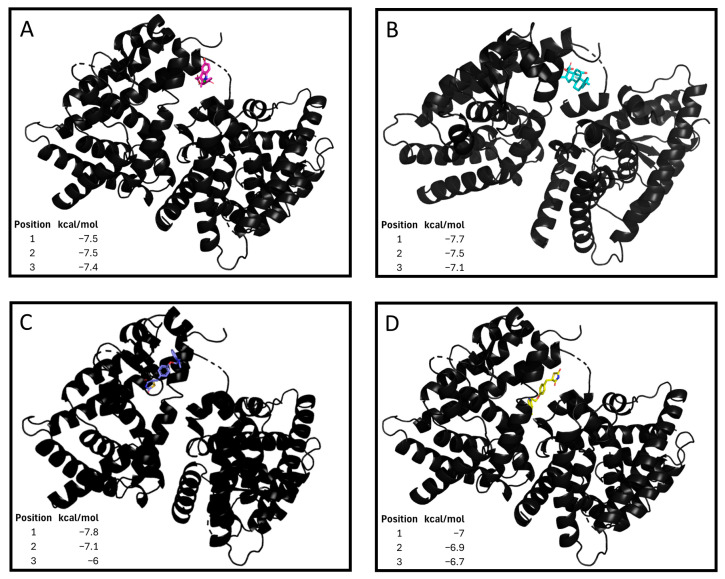
Tritherapy compounds binding affinity to **peroxisome proliferator-activated receptor gamma (PPARG)**. (**A**) Dexamethasone–PPARG molecular docking. Gibbs free energy values (kcal/mol) for the 3 interactions with higher strengths. (**B**) Indomethacin–PPARG molecular docking. Gibbs free energy values (kcal/mol) for the 3 interactions with higher strengths. (**C**) Pioglitazone–PPARG molecular docking. Gibbs free energy values (kcal/mol) for the 3 interactions with higher strengths. (**D**) Rosiglitazone–PPARG molecular docking. Gibbs free energy values (kcal/mol) for the 9 interactions with higher strengths.

PPARG has emerged as a key factor that modulates the osteoblastic–adipogenic equilibrium [20]. Therefore, we sought a repurposed combined therapy to control this balance through the promotion of the adipogenic environment and PPARG signalling. Thus, we propose three compounds: a thiazolidinedione, an oral antidiabetic; dexamethasone, a corticoid that promotes the bone adipogenic environment [21]; and indomethacin, an NSAID. Thiazolidinedione and indomethacin PPARG agonism has been demonstrated previously [22,23]. Nonetheless, the interaction between dexamethasone and PPARG has not been described. When performing a computational approach, dexamethasone (−7.5 kcal/mol), indomethacin (−7.7 kcal/mol), pioglitazone (−7.8 kcal/mol) and rosiglitazone (−7.0 kcal/mol) were robust and had similar binding affinities in different locations of the same region of the receptor (Figure 1). Thus, the value of these drugs in altering the osteoblastogenic–adipogenic balance is increased. As a result, three drugs (thiazolidinedione, indomethacin, and dexamethasone) were combined to create a tritherapy, the effects of which were tested in diverse bone precursor cells as potential treatments for abnormal bone growth.

### 2.2. Osteoblast–Adipocyte Balance Modulation in MSCs (C3H10T1/2)

**Figure 2:**
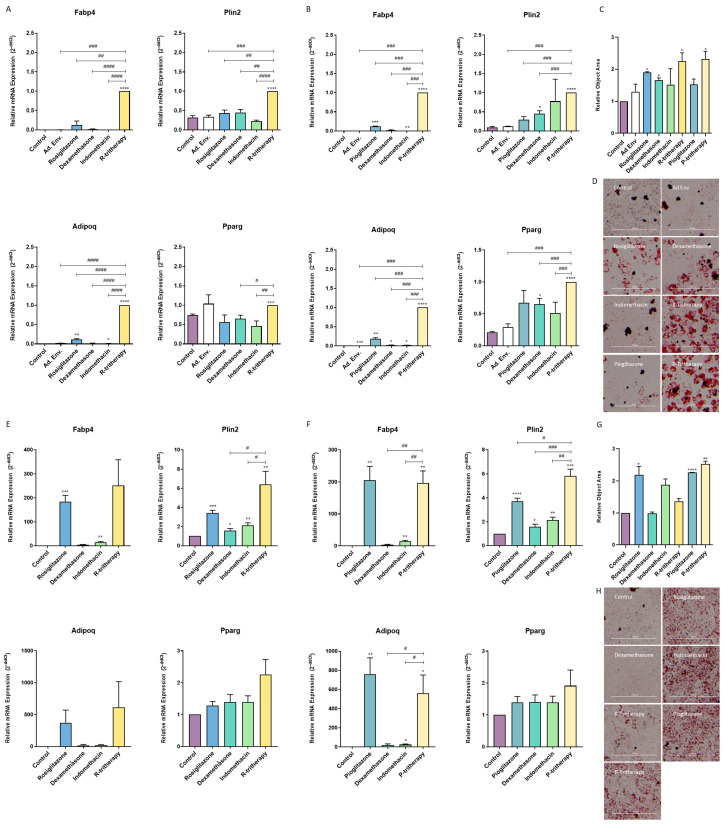
**Tritherapy effects on C3H10T1/2 adipogenesis and osteoblastogenesis.** (**A**) Expression of the adipogenesis marker genes fatty acid-binding protein 4 (Fabp4), perilipin 2 (Plin2), adiponectin (Adipoq) and peroxisome proliferator-activated receptor gamma (Pparg) was measured via RT–PCR in C3H10T1/2 cells treated for 7 days in a proadipogenic environment (culture medium supplemented with insulin growth factor-1 (IGF1), 3-isobutyl-methylxanthine (IBMX) and insulin) with rosiglitazone, dexamethasone, indomethacin, and tritherapy with rosiglitazone (R-tritherapy). # *p* < 0.05, ## *p* < 0.01, ### *p* < 0.001, #### *p* < 0.0001 vs. R-tritherapy. (**B**) Expression of the adipogenesis marker genes Fabp4, Plin2, Adipoq and Pparg, as measured via RT–PCR, in C3H10T1/2 cells treated for 7 days in a proadipogenic environment (culture medium supplemented with IGF1, IBMX and insulin) with pioglitazone, dexamethasone, indomethacin, and tritherapy with pioglitazone (P-tritherapy). ### *p* < 0.001 vs. P-tritherapy. (**C**) Relative Object Area of lipid droplets in C3H10T1/2 cells treated for 7 days in a proadipogenic environment (culture medium supplemented with IGF1, 3-isobutyl-methylxanthine (IBMX) and insulin) with rosiglitazone, dexamethasone, indomethacin, R-tritherapy, pioglitazone and P-tritherapy. (**D**) Lipid droplets Oil Red O staining in C3H10T1/2 cells treated for 7 days in a proadipogenic environment (culture medium supplemented with IGF1, 3-isobutyl-methylxanthine (IBMX) and insulin) with rosiglitazone, dexamethasone, indomethacin, R-tritherapy, pioglitazone and P-tritherapy. Scale bar corresponds to 300 μm. (**E**) Expression of the adipogenesis marker genes Fabp4, Plin2, Adipoq and Pparg, as measured via RT–PCR, in C3H10T1/2 cells treated for 7 days in a proosteoblastogenic environment with rosiglitazone, dexamethasone, indomethacin, and R-tritherapy. # *p* < 0.05 vs. R-tritherapy. (**F**) Expression of the adipogenesis marker genes Fabp4, Plin2, Adipoq and Pparg, as measured via RT–PCR, in C3H10T1/2 cells treated for 7 days in a proosteoblastogenic environment with pioglitazone, dexamethasone, indomethacin, and P-tritherapy. # *p* < 0.05, ## *p* < 0.01, ### *p* < 0.001 vs. P-tritherapy. (**G**) Relative Object Area of lipid droplets in C3H10T1/2 cells treated for 7 days in a proosteoblastogenic environment with rosiglitazone, dexamethasone, indomethacin, R-tritherapy, pioglitazone and P-tritherapy. (**H**) Lipid droplets Oil Red O staining in C3H10T1/2 cells treated for 7 days in a proosteoblastogenic environment with rosiglitazone, dexamethasone, indomethacin, R-tritherapy, pioglitazone and P-tritherapy. Scale bar corresponds to 300 μm. * *p* < 0.05, ** *p* < 0.01, *** *p* < 0.001, **** *p* < 0.0001 versus the control.

The C3H10T1/2 cell line has been described as a proper model to study the balance between osteoblastogenesis and adipogenesis [24]. Rosiglitazone is the thiazolidinedione lead drug; nonetheless, it is currently not employed in clinical practice. Thus, we also tested the effect of pioglitazone, which is the most commonly used thiazolidinedione. Treatment of these cells with tritherapy in a proadipogenic environment for 7 days significantly increased the expression of adipogenic marker genes (Figure 2A,B) and the accumulation of lipids in the cells (Figure 2C,D). For those genes not directly activated by thiazolidinediones, tritherapy had a synergistic effect compared with each individual drug treatment (Figure 2A,B).

Moreover, when C3H10T1/2 differentiated into osteoblasts for 7 days, tritherapy increased Fabp4, Plin2 and Adipoq expression (Figure 2C,D) and the lipid accumulation in the cells (Figure 2G,H). The effect of rosiglitazone on these cells was not different from that of R-tritherapy, including this drug (Figure 2). Consistently, pioglitazone exhibited the same behaviour even though Plin2 expression was increased by P-tritherapy (Figure 2).

### 2.3. Osteoblastogenesis Modulation in a Osteoblastic-like Cell Line (SaOS2)

**Figure 3:**
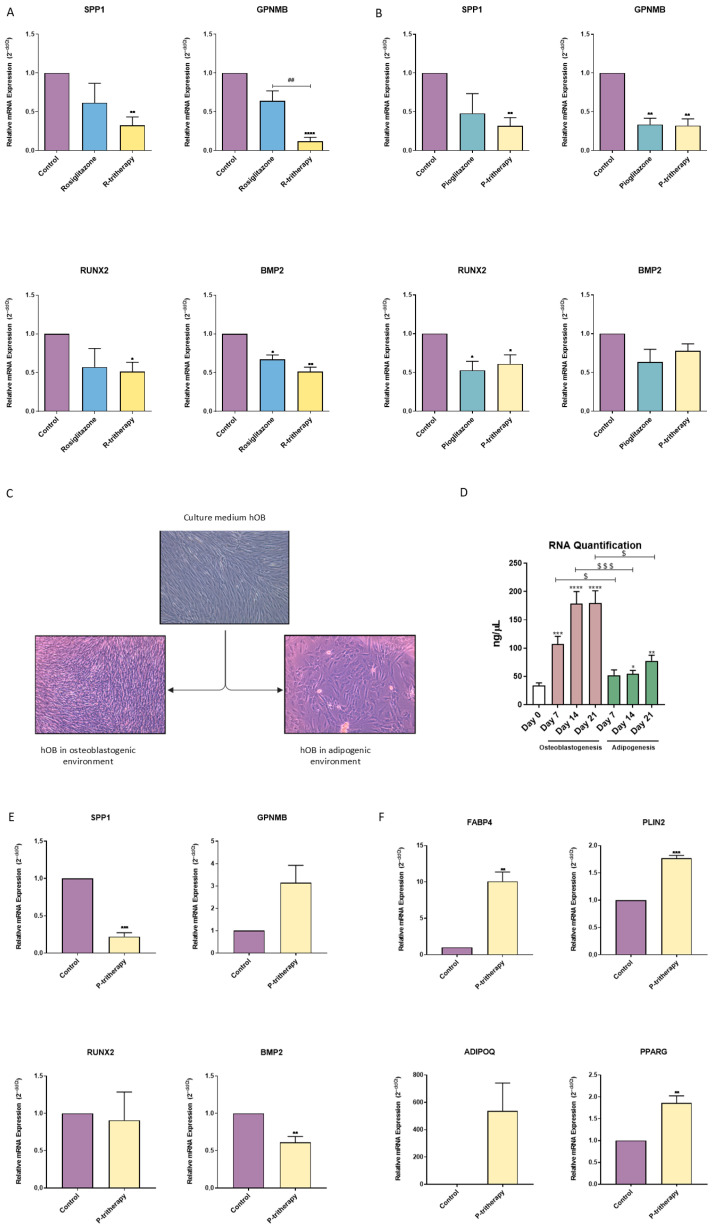
**Tritherapy effects on SaOS2 and human primary preosteoblast-like** (**hOB**) **cell osteoblastogenesis**. (**A**) Expression of the osteoblastogenesis marker genes osteopontin (SPP1), osteoactivin (GPNMB), RUNT-related transcription factor 2 (RUNX2) and bone morphogenic protein 2 (BMP2) was measured via RT–PCR in the SaOS2 osteoblastic-like cell line treated with rosiglitazone and rosiglitazone (R-tritherapy) for 14 days in a proosteoblastogenic environment. * *p* < 0.05, ** *p* < 0.01, **** *p* < 0.0001 versus the control. ## *p* < 0.01, vs. R-tritherapy. (**B**) Expression of the osteoblastogenesis marker genes SPP1, GPNMB, RUNX2 and BMP2, as measured by RT–PCR, in the SaOS2 osteoblastic-like cell line treated with pioglitazone and pioglitazone (P-tritherapy) for 14 days in a proosteoblastogenic environment. * *p* < 0.05, ** *p* < 0.01 versus the control. (**C**) Differences in cellularity between hOBs in normal growth medium (DMEM:F12), an osteoblastogenic environment and an adipogenic environment for 11 days (4×). (**D**) RNA concentration (ng/μL) of hOBs after maintaining osteoblastogenesis and adipogenesis for 21 days. * *p* < 0.05, ** *p* < 0.01, *** *p* < 0.001, **** *p* < 0.0001 versus day 0. $ *p* < 0.05, and $$$ *p* < 0.001 between the differentiation processes. (**E**) Expression of the osteoblastogenesis marker genes SPP1, GPNMB, RUNX2, and BMP2, as measured via RT–PCR, in hOBs treated with P-tritherapy for 7 days in a proosteoblastogenic environment. ** *p* < 0.01, *** *p* < 0.001 versus the control. (**F**) Expression of the adipogenic marker genes fatty acid-binding protein 4 (FABP4), perilipin 2 (PLIN2), adiponectin (ADIPOQ) and peroxisome proliferator-activated receptor gamma (PPARG) was measured via RT–PCR in hOBs treated for 7 days in a proosteoblastogenic environment with P-tritherapy. ** *p* < 0.01, *** *p* < 0.001, versus the control.

Once the tritherapy effect was assessed in the early stages of differentiation, it was also determined in more advanced phases of osteoblastogenesis. After 14 days of SaOS2 osteoblastic differentiation and treatment with thiazolidinediones or tritherapy, R-tritherapy decreased SPP1, GPNMB, RUNX2 and BMP2 expression (Figure 3A), and P-tritherapy decreased SPP1, GPNMB, and RUNX2 expression (Figure 3B). In contrast, rosiglitazone itself only reduced BMP2 expression, and pioglitazone reduced GPNMB and RUNX2 expression (Figure 3A,B).

### 2.4. Preosteoblast-like Cell Growth Modulation

Currently, no therapeutic approach to modulate the balance between osteoblastogenesis and adipogenesis is available. Because adipogenesis and osteoblastogenesis are opposite and mutually regulated processes, we explored the effect of the adipogenic environment on hOB. Figure 3C shows how the proliferation rate of primary hOBs changed when they were exposed to different culture media. The confluence of hOBs cultured in a proadipogenic environment (described as medium supplemented with insulin growth factor-1 (IGF1), 3-isobutyl-methylxanthine (IBMX) and insulin) drastically decreased (Figure 3C). This result was consistent with the reduction in RNA concentrations in hOB cells exposed to adipogenic media for 21 days (Figure 3D).

### 2.5. Osteoblastogenesis–Adipogenesis Balance Modulation in Human Primary Preosteoblastic-like Cells

Considering how scarce and difficult it is to obtain hOB cells, only P-tritherapy, including commercialised thiazolidinedione (pioglitazone), was tested. The effects of P-tritherapy were evaluated in hOB cells during 7 days of osteoblastogenesis. Despite the patients’ intrinsic variability (as observed in GPNMB), P-tritherapy significantly diminished the expression of the osteoblastic marker genes SPP1 and BMP2 (Figure 3E). Moreover, it increased the expression of the adipogenic marker genes FABP4, PLIN2, and PPARG (Figure 3F). These modulations confirmed the potential of P-tritherapy as an osteoblastogenesis modulator.

### 2.6. In Vitro Regulation of Endochondral Ossification

**Figure 4:**
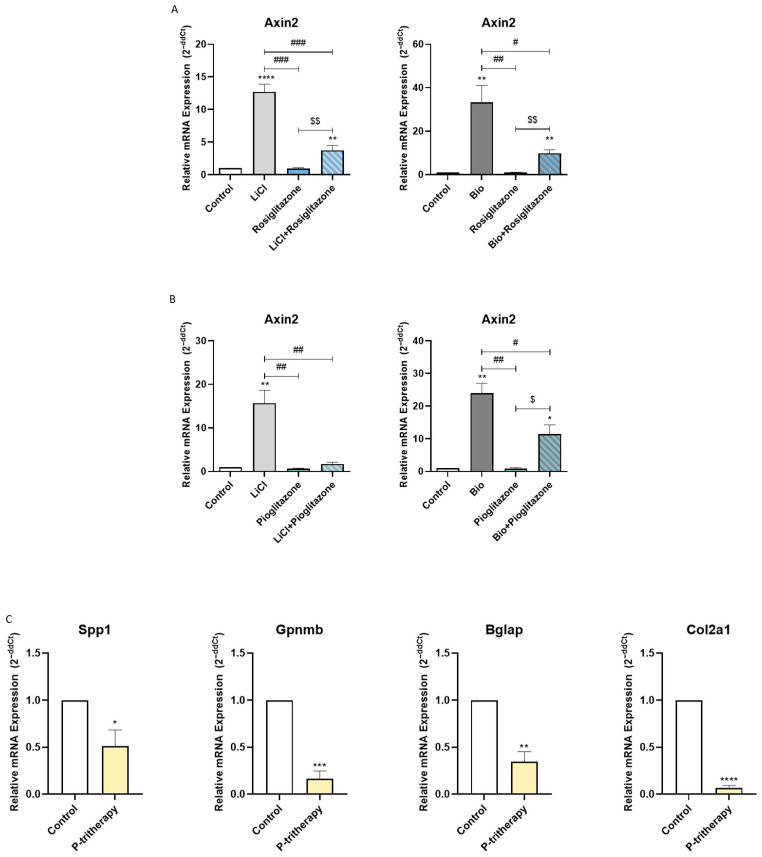
**Tritherapy effects on endochondral ossification.** (**A**) Expression of the WNT effector gene Axin2, as measured by RT–PCR, in the prechondrogenic ATDC5 cell line with or without WNT pathway stimuli (LiCl, BIO) and rosiglitazone. $$ *p* < 0.01between the rosiglitazone-treated groups. # *p* < 0.05, ## *p* < 0.01, ### *p* < 0.001 vs. the stimuli treated. (**B**) Expression of the WNT effector gene Axin2, as measured by RT–PCR, in the prechondrogenic ATDC5 cell line with or without WNT pathway stimuli (LiCl, BIO) and pioglitazone. $ *p* < 0.05 between the pioglitazone-treated groups. # *p* < 0.05, ## *p* < 0.01 vs. the stimuli treated. (**C**) Expression of the osteoblastic and chondrogenic marker genes osteopontin (Spp1), osteoactivin (Gpnmb), osteocalcin (Bglap) and collagen-2-α-1 (Col2a1) was measured via RT–PCR in an endochondral ATDC5 cell line model treated with P-tritherapy for 28 days. * *p* < 0.05, ** *p* < 0.01, *** *p* < 0.001, **** *p* < 0.0001 versus the control.

Heterotopic ossification can occur from intramembranous or endochondral ossification [25]. Endochondral bone growth is closely related to WNT pathway activation [26]. Hence, considering that the activation of PPARG is related to WNT pathway modulation [27], we decided to study whether thiazolidinediones, PPARG agonists, affect WNT signalling activation in chondrogenic cells. As shown in Figure 4A,B, thiazolidinediones (rosiglitazone and pioglitazone) significantly inhibited the expression of Axin2, a WNT pathway marker gene induced by two well-known WNT pathway activators (LiCl and BIO).

Accordingly, we decided to evaluate whether P-tritherapy modulated the ossification of chondrogenic precursor cells. As shown in Figure 4C, P-tritherapy diminished the expression of Spp1, Gpnmb, Bglap and Col2a1 after 28 days of ATDC5 endochondral ossification in an in vitro model.

### 2.7. Osteoblast–Adipocyte Balance Modulation in Heterotopic Ossification

**Figure 5:**
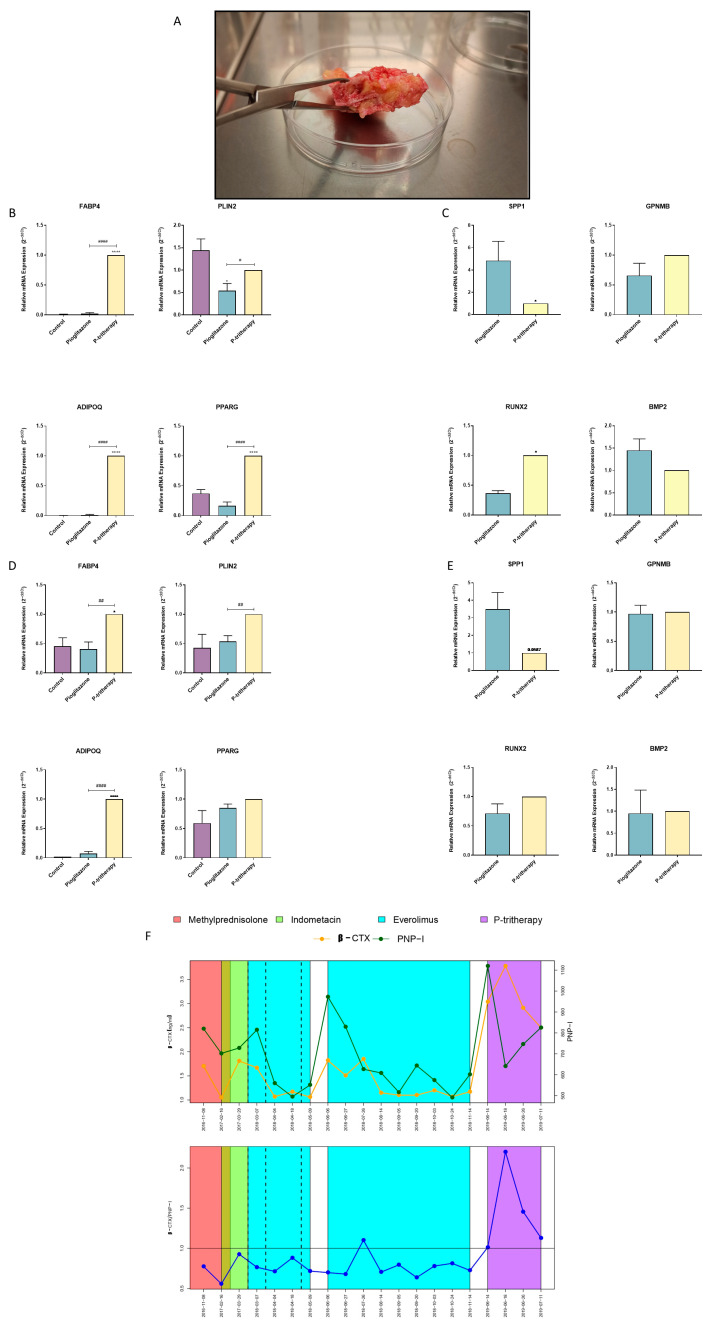
Tritherapy in heterotopic ossification human primary preosteoblast-like cells (HO hOB) and in a POH patient. (**A**) POH bone growth. (**B**) Expression of the adipogenic marker genes fatty acid-binding protein 4 (FABP4), perilipin 2 (PLIN2), adiponectin (ADIPOQ) and peroxisome proliferator-activated receptor gamma (PPARG) was measured via RT–PCR in HO hOBs from a patient with acquired HO who was treated for 7 days in a proadipogenic environment with tritherapy with pioglitazone (P-tritherapy). * *p* < 0.05, **** *p* < 0.0001 versus the control. # *p* < 0.05, #### *p* < 0.0001 vs. P-tritherapy. (**C**) Expression of the osteoblastogenesis marker genes osteopontin (SPP1), osteoactivin (GPNMB), RUNT-related transcription factor 2 (RUNX2) and bone morphogenic protein 2 (BMP2) was measured via RT–PCR in HO hOBs from a patient with acquired HO treated with pioglitazone and P-tritherapy for 7 days in a proadipogenic environment. * *p* < 0.05, versus pioglitazone. (**D**) Expression of the adipogenic marker genes FABP4, PLIN2, ADIPOQ, and PPARG in POH hOBs from a patient with congenital HO (POH) treated for 7 days in a proadipogenic environment with P-tritherapy was measured via RT–PCR. * *p* < 0.05, **** *p* < 0.0001 versus the control. ## *p* < 0.01, #### *p* < 0.0001 vs. P-tritherapy. (**E**) Expression of the osteoblastogenesis marker genes SPP1, GPNMB, RUNX2, and BMP2, as measured by RT–PCR, in POH hOBs from a patient with congenital HO (POH) treated with pioglitazone and P-tritherapy for 7 days in a proadipogenic environment. (**F**) Serum procollagen type 1 N-terminal propeptide (PNPI) and β C-terminal cross-linked telopeptide of type I collagen (βCTX) levels (green and orange line respectively) and ratio (blue line) in a POH patient treated with methylprednisolone, indomethacin, everolimus, and P-tritherapy from 8 November 2016 to 11 July 2019.

Given the results obtained with this tritherapy, we validated its effect in vitro on a rare case of POH with a nonfunctional guanine nucleotide-binding protein (G protein) and an alpha stimulating activity (GNAS) mutation that induced extreme abnormal bone growth in soft tissues [28]. To provide a pharmacological tool to prevent relapse after surgical removal of bone mass, the effects of P-tritherapy were tested in primary human acquired HO cells and in cells from POH patients to compare the effects of P-tritherapy. As shown in Figure 5A, the ectopic POH bone grew embedded in the adipose tissue. Accordingly, a P-tritherapy test was performed in an adipogenic environment to mimic the physiopathology of this disease.

As in skeletal bone-derived hOB cells, the ability of P-tritherapy to inhibit osteoblasts and promote adipocytic phenotypes in acquired HO hOB cells was determined (Figure 5B,C). In these cells, P-tritherapy decreased SPP1 expression and increased RUNX2 expression (Figure 5C). Moreover, P therapy strongly induced an adipogenic phenotype through the induction of the expression of the adipogenic marker genes FABP4, ADIPOQ, and PPARG (Figure 5B).

We obtained similar results in POH-treated hOB cells. P-tritherapy also decreased the expression of SPP1, and the expression of FABP4, PLIN2 and ADIPOQ was significantly greater than the induction of pioglitazone itself, indicating the promotion of the adipogenic phenotype (Figure 5D,E).

### 2.8. POH Clinical Management with Tritherapy

The POH patient described above was previously treated with the available HO therapeutic repertoire, as well as with the active molecules suggested in the literature. However, none of these drugs were able to reverse the relationship between serum procollagen type 1 N-terminal propeptide (PNPI) and β C-terminal cross-linked telopeptide of type I collagen (βCTX) (Figure 5F). Therefore, pathological bone remodelling was maintained [28]. Considering the literature evidence supporting each tritherapy component individually, as well as the P-tritherapy activity on the patient’s primary cells, P-tritherapy administration was proposed to avoid recurrence after ossification excision. The doses and administration plan agreed with the paediatric medical service (pioglitazone: 0.5 mg/kg/day; dexamethasone: 0.08 mg/kg/day; indomethacin: 25 mg every 12 h), which was initiated on the day of surgery. After 4 days, dexamethasone withdrawal was initiated due to patient comorbidities, ending with a bitherapy (pioglitazone and indomethacin) for 27 days. Owing to the patient’s evolution and magnitude of POH, it was impossible to determine the effect of P-tritherapy on plaque recurrence. Nonetheless, as shown in Figure 5F, following 4 days of P-tritherapy treatment, βCTX expression increased, and PNPI expression decreased. These changes inverted the relationship between both bone markers, indicating a change in the remodelling dynamics. This ratio of balance to bone catabolism was maintained during P-tritherapy withdrawal. These data support the use of P-tritherapy as a treatment to prevent abnormal bone anabolism.

## 3. Discussion

In this work, we propose three-drug combination on the basis of their individual binding affinities with the PPARG receptor. This tritherapy aimed at treating and preventing HO was able to modulate the osteoblastogenic–adipogenic balance toward adipogenesis in different cell models. It also diminished the osteoblastic phenotype in intramembranous and endochondral ossification in vitro. Moreover, in a clinical case of POH, P-tritherapy (following validation in acquired and congenital HO primary osteoblasts) inverted the pathological serum bone turnover marker ratio. Thus, tritherapy could be a new therapeutic tool to manage HO.

Kaplan et al. proposed four key principles for the prevention and treatment of HO: the regulation of the participating signalling pathways, the control of HO-associated inflammation, the modulation of osteoprogenitors in those tissues with potential for ossificans, and the control of the pro-ossification environment [7]. Considering these principles, our group searched for a repurposed therapeutic tool able to encompass them all and, therefore, control HO-associated abnormal bone growth. Accordingly, we designed a tritherapy composed of thiazolidinediones (rosiglitazone or pioglitazone), PPARG agonists [22], and two anti-inflammatories. How anti-inflammatory agents affect proper MSC osteoblastogenesis has been described [23,29]. Thus, the corticoid dexamethasone and the NSAID indomethacin were selected. Thiazolidinediones have demonstrated PPARG agonism [22], as does indomethacin [23], which has been previously used in HO treatment [12]. In this work, we demonstrate that dexamethasone also has a binding affinity for the PPARG receptor in different locations in the same region and has a free Gibbs energy comparable to that of the described agonists, the thiazolidinediones. Notably, the four drug binding sites are close but different, suggesting a hypothetical synergistic effect. These results reinforce the anti-osteoblastogenic and proadipogenic potential of dexamethasone and the tritherapy drug combination.

The combination of each thiazolidinedione with dexamethasone and indomethacin synergistically altered the regulation of the balance between osteoblastogenesis and adipogenesis in MSCs (C3H10T1/2), resulting in an adipogenic profile. It has been proposed that adipogenesis is a more spontaneous process than is osteoblastogenesis [30]. Therefore, its promotion could be considered more practical than osteoblastogenesis inhibition with respect to balance modulation. In fully differentiated osteoblasts derived from a cell line and human primary hOBs, R- and P-tritherapy inhibited the osteoblastic phenotype. Additionally, in primary cells, R- and P-tritherapy promoted the adipogenic phenotype. SPP1 has been quoted as a HO key protein [4]. Its lack has been proven to facilitate adipogenesis [5], and one of its roles is to promote fibrosis through the TGFβ pathway, which is one of ossification prior stages [6]. Considering these roles, SPP1 expression diminis in tritherapy-treated cells (SaOS2 and hOB) supported the antiosteoblastogenic effects of the treatment.

Individually, the effects of tritherapy components on endochondral ossification are not fully clear. Thiazolidinediones have been shown to impair endochondral ossification through an increase in adipogenic tissue [31], but their effect on chondrogenic marker genes in this process is yet unknown. The effect of dexamethasone is not clear; however, it affects angiogenesis, a key step in endochondral ossification [32]. The effect of indomethacin on this ossification depends on the stage in which it is administered [33]. In this work, we demonstrated how the combination of thiazolidinediones, dexamethasone and indomethacin was able to modulate both osteogenic and chondrogenic marker genes, thus impairing endochondral ossification. This effect could be exerted, in part, through WNT pathway inhibition by PPARG agonists. A negative relationship between PPARG and this pathway has been described [27]. Since the three compounds of the tritherapy have demonstrated PPARG binding affinity, the tritherapy effect could occur through this pathway modulation, which is key to endochondral ossification [26]. In support of these findings, we observed that PPARG agonism diminished WNT pathway activation in chondrocytes.

Considering POH as an orphan disease and aiming to provide new insights from bench to bedside in a clinical case of this disease [34], P-tritherapy was proposed as a treatment to prevent ossification relapse following bone removal. Individual anti-inflammatory treatment was dismissed since it had already been used without significant results [28]. Before the clinical administration of P-tritherapy, its effect on HO hOB from acquired HO and POH hOB from the patients themselves was tested in vitro. As previously described [8], in this concrete case, ossifications grow in soft tissues in a subcutaneous adipose environment. Therefore, the experiments were performed in a proadipogenic environment to mimic the tissue context where P-tritherapy should work. The obtained results were not as strong as those in hOB. This could be due to the greater osteogenic potential of hOB HO cells [35]. Nonetheless, P-tritherapy augmented adipogenesis marker gene expression, increased spontaneous MSC differentiation, and decreased the expression of the key HO marker gene SPP1 compared to pioglitazone alone. The induction of RUNX2 expression in HO hOBs by P-tritherapy could seem controversial. RUNX2 inhibition in HO models has been successful in diminishing disease progression [36,37]. Nonetheless, in a normal ossification process, RUNX2 expression must decrease to complete osteoblast maturation [38]. These findings suggest that the tritherapy-induced impairment of the final differentiation of osteoblasts is due to the decrease in RUNX2 expression. As an alternative, the increase in RUNX2 expression caused by P-tritherapy in HO hOBs could be due to a compensatory mechanism from the cells, in contrast to the strong adipogenic potential of tritherapy. Nonetheless, neither in POH HO hOB nor in healthy hOB was this significant increase observed. Thus, as with SPP1 expression, this effect could be due to their greater osteogenic potential [35]. Once assessed in vitro, the effectiveness of clinical P-tritherapy administration on the osteoblast–osteoclast balance was confirmed. The rupture of the relationship between the clinical bone remodelling markers PNPI and βCTX [39] for the first time at 3 years of follow-up confirmed that the proposed P-tritherapy not only controlled the four key points proposed by Kaplan et al. [7] but also further balanced bone remodelling toward resorption.

Alternative or complementary approaches to preventing HO calcification involve taking advantage of the body’s endogenous calcification inhibitors as matrix Gla protein (MGP), progressive ankylosis protein homologue (ANKH) or others [40]. Therapeutic strategies to enhance these pathways could control HO calcification processes from an approach different from adipogenesis promotion. Thus, it can be considered that a combination of both strategies could be beneficial. The merge of adipogenesis promotion and osteoblastogenesis inhibition with internal calcification-inhibitory mechanisms could provide a multifaceted strategy to prevent abnormal bone growth.

After we evaluated the effects of tritherapy, we identified several clinical limitations. It is conceivable that systemic tritherapy administration could have adverse effects on healthy bone. The majority of HO cases are localised and/or secondary to surgical removal [41]. In these cases, the tritherapy administration would be planned to be limited to the time needed to block the osteoblastogenesis process (weeks) and administered locally to prevent recidivism. In these administration patterns, the harmful effect on healthy bone should be diminished. With respect to congenital HO, the severity of each clinical case should be evaluated by clinicians to establish the benefit–risk ratio. In any case, the systemic regimen should be rigorously evaluated prior to patient application to avoid bone secondary effects of some of the drugs comprising tritherapy.

## 4. Materials and Methods

### 4.1. Reagents

Basic medium components (Dulbecco’s modified Eagle’s medium (DMEM), DMEM supplemented with HAM’S-F12 (DMEM-F12), penicillin/streptomycin, L-glutamine and foetal bovine serum (FBS)) were obtained from Sigma–Aldrich (St. Louis, MO, USA). Unless otherwise specified, all reagents were purchased from Sigma–Aldrich. IGF1 was obtained from PeproTech, Inc. (Rocky Hill, CT, USA).

The ATDC5 murine chondrogenic cell line was obtained from RIKEN Cell Bank (Tsukuba, Japan). Dr. Pardo (IDIS Institute) selflessly donated C3H10T1/2, a murine mesenchymal stem cell line. SaOS2, a human osteoblast-like cell line, was purchased from CLS (Eppelheim, Germany).

### 4.2. Docking Studies

The 3D structures of rosiglitazone, pioglitazone, indomethacin, and dexamethasone were obtained from the RCSB Protein Data Bank (PDB). The docking analysis was performed as in previous works [42].

### 4.3. Cell Culture and Differentiation

Total knee replacement surgery is the source of human primary preosteoblast-like cells (hOBs) from aged women (between 60 and 75 years old). Bone ossification exeresis in acquired ossification patients (hip ossification) and POH patients (scapular, tummy, and butt ossifications) was the source of heterotopic ossification hOBs (HO hOB and POH hOB, respectively). The protocol (CAEIG-2016/258) was approved by the Ethics Committee for Research in the Santiago-Lugo area. All patients signed and provided informed consent. Current guidelines and regulations were followed to perform all the processes.

C3H10T1/2 cells were maintained in DMEM supplemented with 10% FBS, 100 U/mL penicillin/streptomycin, and 4 mM L-glutamine. A total of 10^4^ cells were seeded in each well of a 24-well plate for differentiation. Six hours after seeding, the differentiation process was initiated. Adipogenesis and osteoblastogenesis were performed for 7 days as previously described to reach adipogenic profile [24]. The proposed tritherapy was added to the differentiation medium individually and concomitantly at the following concentrations: thiazolidinedione 2 μM rosiglitazone (R-tritherapy) or 10 μM pioglitazone (P-tritherapy), 1 μM dexamethasone, and 60 μM indomethacin. The adipogenic environment was generated with adipocyte basal medium (containing IGF1, insulin, and IBMX).

SaOS2 cell growth medium included DMEM-F12, 5% FBS, 100 U/mL penicillin/streptomycin, and 2 mM L-glutamine. Osteoblast differentiation was initiated once 1.3 × 10^5^ seeded cells in 6-well plates reached confluence. Growth medium supplemented with 50 µg/mL ascorbic acid was used to induce differentiation for 14 days to reach osteoblastogenic profile as in previous works [24]. Tritherapy was added as previously described for C3H10T1/2.

hOB and HO hOB were cultured in DMEM-F12 supplemented with 4 mM glutamine, 200 U/mL penicillin/streptomycin, and 10% FBS. The differentiated seeds consisted of 45·10^3^ cells per well in a 24-well plate. After 24 h of adipocyte and osteoblast differentiation, tritherapy treatment was initiated as previously described for the C3H10T1/2 cell line.

The ATDC5 maintenance medium consisted of DMEM/F12 supplemented with 5% FBS, 100 UI/mL penicillin/streptomycin, and 4 mM L-glutamine. A total of 10^5^ cells were seeded in a 12-well plate to assess Wingless-related integration site (WNT) inhibition by thiazolidinediones. Six hours after seeding, the cells were starved with serum-free medium, and 18 h later, the cells were treated with WNT inductors (LiCl 20 mM, BIO 1 µM, and Wnt3a 100 ng/mL (TebuBio, Le Perray-en-Yvelines, France)) with/without thiazolidinediones for 8 h. To establish the endochondral ossification model, 10^3^ cells were seeded in a p6 plate. These chondrocytes were differentiated into hypertrophic chondrocytes for 14 days with ATDC5 differentiation medium supplemented with 17.48 μg/mL insulin. Then, 5 mM β-glycerol-2-phosphate, 50 μg/mL ascorbic acid-2-phosphate and 10 nM dexamethasone were added for 14 days. P-tritherapy was added simultaneously and continuously during the 28 days of the experiment.

### 4.4. Gene Expression Analysis

Eight hours after ATDC5 cells were treated with WNT inducers and thiazolidinediones, the cells were lysed with Tri-Reagent (Sigma–Aldrich, St Louis, MO, USA). E.Z.N.A. A total RNA kit I (Omega, Bio-Tek, Inc., Norcross, GA, USA) was used to isolate cellular RNA following the manufacturer’s guidelines. cDNA was obtained with a high-capacity cDNA reverse transcription kit (Applied Biosystems, Life Technologies, Grand Island, NY, USA). 

Axin2 WNT-induced gene marker mRNA expression was determined by real-time PCR using Bio-Rad MasterMix (Hercules, CA, USA) and primers from Sigma–Aldrich (St. Louis, MO, USA) (Table 1).

After 7 days of adipocyte differentiation and 14 days of osteoblastic differentiation, cell lysis, RNA extraction and cDNA retrotranscription were performed as described previously. Fatty acid-binding protein 4 (FABP4), perilipin 2 (PLIN2), adiponectin (ADIPOQ), peroxisome proliferator-activated receptor gamma (PPARG), osteopontin (SPP1), osteoactivin (GPNMB), runt-related transcription factor-2 (RUNX2), bone morphogenic protein 2 (BMP2), osteocalcin (Bglap), and collagen-2 α1 (Col2a1) were considered marker genes.

### 4.5. Oil Red O Staining

Lipid droplets staining with Oil Red O followed the same protocols as in previous works [24]. Object area of the lipid dropplets was measured with Gen 5 software (BioTek, Inc., Winooski, VT, USA) using a mask for red colour, object size range 1–40 μm.

### 4.6. Clinical Administration

P-tritherapy off-label administration to a POH patient was approved by the Santiago University Clinical Hospital Committee. The doses and administration plan agreed with the paediatric medical service (pioglitazone: 0.5 mg/kg/day; dexamethasone: 0.08 mg/kg/day; indomethacin: 25 mg every 12 h), which was initiated on the day of surgery. After 4 days, dexamethasone withdrawal was initiated due to patient comorbidities, ending with a bitherapy (pioglitazone and indomethacin) for 27 days.

### 4.7. Statistical Analysis

The data are presented as the means ± standard errors of the means (SEMs) of at least three independent experiments. Significant differences were calculated via a parametric or nonparametric test according to the sample type. All the statistical analyses were performed via Prism software 10 (GraphPad Software Inc., La Jolla, CA, USA), and *p* < 0.05 was considered significant.

Some adipogenic genes (as Adipoq) are inducible, thus, they are not always equally expressed at Control point. Hence, tritherapy normalisation avoided data loss and variability in adipogenic environment experiments. This adjustment was not necessary in the rest of the experiments.

## 5. Conclusions

HO is a process by which bone tissues grow outside the skeleton. It can be congenital or acquired, and its incidence varies enormously in terms of its origin. This abnormal bone growth can occur from intramembranous ossification and from endochondral ossification, making treatment of this pathology difficult. Currently, there is no effective, nonaggressive treatment for heterotopic ossification or preventive therapeutic tool. In this work, we propose a tritherapy comprising a thiazolidinedione, dexamethasone, and indomethacin to treat and prevent HO through the regulation of osteoblastogenic–adipogenic balance, the osteoblastic phenotype, and endochondral ossification processes.

## 6. Patents

The authors A.A-P., E.F-T., M.G-F., A.A.J.-M., and R.G. declare that the results described herein are patented (Patent number: US12251376B2), with this patent held by Servicio Galego de Saúde (SERGAS).

## Figures and Tables

**Table 1:** Primer sequences for real-time PCR.

Gene	Forward Primer	Reverse Primer
Axin2	AAGATCACAAAGAGCCAAAG	GAAAAAGTAGGTGACAACCAG
FABP4	CAAGAGCACCATAACCTTAG	CTCGTTTTCTCTTTATGGTGG
Fabp4	GTAAATGGGGATTTGGTCAC	TATGATGCTCTTCACCTTCC
PLIN2	GTTCACCTGATTGAATTTGC	GAGGTAGAGCTTATCCTGAG
Plin2	ATAAGCTCTATGTCTCGTGG	GCCTGATCTTGAATGTTCTG
ADIPOQ	GGTCTTATTGGTCCTAAGGG	GTAGAAGATCTTGGTAAAGCG
Adipoq	CCACTTTCTCCTCATTTCTG	CTAGCTCTTCAGTTGTAGTAAC
PPARG	AAAGAAGCCAACACTAAACC	TGGTCATTTCGTTAAAGGC
Pparg	AAAGACAACGGACAAATCAC	GGGATATTTTTGGCATACTCTG
SPP1	GACCAAGGAAAACTCACTAC	CTGTTTAACTGGTATGGCAC
Spp1	GGATGAATCTGACGAATCTC	GCATCAGGATACTGTTCATC
GPNMB	CAGATCAGATTCCTGTGTTTG	ACAGTATGATTGGTGGAAAC
Gpnmb	CTCTTTAATGCCTACTGGTTAC	GCCATATCTGTTTATTCGGC
RUNX2	AAGCTTGATGACTCTAAACC	TCTGTAATCTGACTCTGTCC
Runx2	ACAAGGACAGAGTCAGATTAC	CAGTGTCATCATCTGAAATACG
BMP2	TCCACCATGAAGAATCTTTG	TAATTCGGTGATGGAAACTG
Bglap	ACCATGAGGACCATCTTTC	GGACATGAAGGCTTTGTC
Col2a1	GCGATGACATTATCTGTGAAG	TATCTCTGATATCTCCAGGTTC

Mouse Axin2 WNT induction gene (Axin2), human fatty acid-binding protein 4 (FABP4), mouse fatty acid-binding protein 4 (Fabp4), human perilipin 2 (PLIN2), mouse perilipin 2 (Plin2), human adiponectin (ADIPOQ), mouse adiponectin (Adipoq), human peroxisome proliferator-activated receptor gamma (PPARG), mouse peroxisome proliferator-activated receptor gamma (Pparg), human osteopontin (SPP1), mouse osteopontin (Spp1), human osteoactivin (GPNMB), mouse osteoactivin (Gpnmb), human runt-related transcription factor-2 (RUNX2), mouse runt-related transcription factor-2 (Runx2), human bone morphogenic protein 2 (BMP2), mouse osteocalcin (Bglap), and mouse collagen-2 α1 (Col2a1).

## Data Availability

The datasets used and/or analysed during the current study are available from the corresponding author on reasonable request. The data are not publicly available due to the patent of a part of the article’s results (Patent number: US12251376B2).

## Abbreviations

The following abbreviations are used in this manuscript:

HO	Heterotopic ossification
ADIPOQ	Human adiponectin
Adipoq	Mouse adiponectin
ANKH	Progressive ankylosis protein homologue
Bglap	Mouse osteocalcin
BMP2	Human bone morphogenic protein 2
Col2a1	Mouse collagen-2 α1
DMEM	Dulbecco’s modified Eagle’s medium
FABP4	Human fatty acid-binding protein 4
Fabp4	Mouse fatty acid-binding protein 4
FBS	Foetal bovine serum
FOP	Fibrodysplasia ossificans progressive
GPNMB	Human osteoactivin
Gpnmb	Mouse osteoactivin
hOBs	human primary preosteoblast-like cells
IBMX	3-isobutyl-methylxanthine
IGF1	Insulin growth factor-1
MGP	Matrix Gla protein
MSCs	Mesenchymal stem cells
NSAIDs	Nonsteroid anti-inflammatory drugs
PDB	Protein Data Bank
PLIN2	Human perilipin 2
Plin2	Mouse perilipin 2
POH	Progressive osseous heteroplasia
PPARG	Human peroxisome proliferator-activated receptor gamma
Pparg	Mouse peroxisome proliferator-activated receptor gamma
P-tritherapy	Tritherapy comprising pioglitazone, dexamethasone, and indomethacin
RT-qPCR	Real time quantitative PCR
R-tritherapy	Tritherapy comprising rosiglitazone, dexamethasone, and indomethacin
RUNX2	Human runt-related transcription factor-2
Runx2	Mouse runt-related transcription factor-2
SEMs	Standard Errors of the Means
SPP1	Osteopontin
Spp1	Mouse osteopontin
TGFβ	Transforming growth factor β
WNT	Wingless-related integration site
